# Safety and efficacy of His-Purkinje system pacing in the treatment of patients with atrial fibrillation and heart failure: a systematic review and meta-analysis

**DOI:** 10.3389/fcvm.2023.1233694

**Published:** 2023-09-13

**Authors:** Lin Guan, Chuanhe Wang, Xueqing Guan, Gong Cheng, Zhijun Sun

**Affiliations:** Department of Cardiology, Shengjing Hospital of China Medical University, Shenyang, China

**Keywords:** atrial fibrillation, heart failure, His-Purkinje system pacing, His bundle pacing, left bundle branch pacing, meta-analysis

## Abstract

**Aim:**

To evaluate the safety and efficacy of the His-Purkinje system pacing (HPCSP) in the treatment of individuals with atrial fibrillation (AF) complicated by heart failure (HF).

**Methods:**

The PubMed, Cochrane Library, Web of Science, and Embase databases were searched through September 1, 2022. The literature was initially screened based on the inclusion and exclusion criteria. The baseline characteristics of the subjects, implantation success rate, New York Heart Association (NYHA) classification, left ventricular ejection fraction (LVEF), left ventricular end-diastolic diameter (LVEDd), QRS duration, pacing threshold, and impedance were extracted and summarized; statistical analysis was performed using RevMan 5.3 software.

**Results:**

In all, 22 articles were included, involving 1,445 patients. Compared to biventricular pacing (BiVP), HPCSP resulted in improved cardiac function, including increased ejection fraction (MD = 5.69, 95% CI: 0.78–10.60, *P* = 0.02) and decreased LVEDd (MD = −3.50, 95% CI: −7.05–0.05, *P* = 0.05). It was also correlated with shorter QRS duration (MD = −38.30, 95% CI: −60.71–−15.88, *P* < 0.01) and reduced all-cause mortality and rehospitalization events (RR = 0.72, 95% CI: 0.57–0.91, *P* < 0.01) in patients. Left bundle branch pacing (LBBP) lowered the pacing threshold (MD = 0.47; 95% CI: 0.25–0.69; *P* < 0.01), and there was no statistical difference in the rate of endpoint events when comparing these two physiologic pacing modalities (RR = 1.56, 95% CI: 0.87–2.80, *P* = 0.14).

**Conclusion:**

The safety and efficacy of HPCSP in patients with AF and HF were verified in this meta-analysis. HPCSP can reverse cardiac remodeling and has great clinical application value. Relatively speaking, His-bundle pacing (HBP) can maintain better ventricular electro-mechanical synchronization, and the pacing parameters of LBBP are more stable.

**Systematic Review Registration:**

PROSPERO (CRD42022336109)

## 1. Introduction

Epidemiological studies have shown that heart failure (HF) and atrial fibrillation (AF) are two common cardiovascular diseases (CVD), with an increasing incidence worldwide (1). The two diseases act synergistically and causally, i.e., one of these diseases can lead to the development or exacerbation of another, ultimately having a synergistic negative effect on cardiovascular health and quality of life (2).

In recent years, the choice of rhythm and ventricular rate control in patients with AF combined with HF has been an important issue of active discourse by experts globally. Currently, some guidelines confirm that there is a certain risk of recurrence after catheter ablation; moreover, catheter ablation may not be an appropriate choice to maintain sinus rhythm in some patients with long-range persistent AF, abnormal left atrial enlargement, and low left ventricular ejection fraction (3). With the accumulation of evidence-based medicine, several trials, including the APAF-CRT study have found that the treatment strategy of AV node ablation combined with cardiac resynchronization therapy **(**CRT) can significantly reduce all-cause mortality in patients, and it can be a preferable option for patients with refractory or high-recurrent AF combined with HF (4). However, in actual clinical application, 30% of patients still do not respond, and the inaccessibility of electrodes due to target vessel malformation is among the main reasons affecting the success rate of CRT (5, 6). Moreover, this pacing mode disturbs the normal sequence of electrical conduction excitation, increases the QRS duration, and even causes partial loss of the original synchronization, which poses a potential risk to patients (7).

His-Purkinje system pacing, as a more physiological pacing mode, is conducted through its own His-Purkinje fiber system, mimicking the normal cardiac electrical conduction sequence of activation (8–11). Several small clinical studies have provided evidence for the efficacy and safety of AV node ablation combined with HPSCP in patients with HF combined with AF, while some large prospective studies are underway (12).

In 2017, Huang et al. demonstrated the safety and stability of permanent His-bundle pacing (HBP) in a group of patients with heart failure and a narrow QRS combined with atrial fibrillation who underwent AVN ablation (13). After a median follow-up time of 20 months, there were significant improvements in New York Heart Association (NYHA) classification, left ventricular ejection fraction (LVEF), and left ventricular end-diastolic diameter (LVEDd), reversing LV remodeling and improving cardiac function in patients with persistent AF even with well-controlled ventricular rates, delaying the progression of heart failure, and reducing rehospitalization and mortality rates. Vijayaraman et al. demonstrated that this treatment modality can improve cardiac function even in patients with poorly controlled ventricular rates (14). Regardless of the ventricular rate control in patients with AF, AV node ablation plus HPCSP is a safe and effective treatment that can significantly improve patient symptoms (15).

Since most of the current studies were single-center, small-sample studies, our study aimed to meta-analyze all relevant clinical studies to increase the sample size and further explore the therapeutic effects and adverse outcomes of HPCSP in the treatment of patients with HF and AF.

## 2. Methods

### 2.1. Search strategy

Two reviewers independently conducted an all-encompassing search of the PubMed, Embase, Web of Science, and Cochrane Library databases, which were restricted to full-text English documents published before September 1, 2022.

The search terms used were {[His bundle pacing (Title/Abstract)] OR [left bundle branch pacing (Title/Abstract)]} AND [atrial fibrillation (Title/Abstract)]. We also conducted a manual search to achieve a comprehensive search.

### 2.2. Study selection

Two investigators used relevant literature management software to screen articles that met the following inclusion criteria: (1) patients with AF and HF with HPCSP indications, aged ≥ 18 years; (2) Study type: randomized controlled study (RCT), prospective or retrospective cohort study (3) Interventions: permanent pacemaker implantation and the pacing mode is HPCSP. (4) Outcomes: Implantation success rate, New York Heart Association (NYHA) classification, left ventricular ejection fraction (LVEF), left ventricular end-diastolic diameter (LVEDd), QRS duration, pacing threshold, impedance, complications, and endpoint events (HF rehospitalization and mortality). The exlusion criteria was: (1) animal studies, reviews, case reports, meta-analyses, conference abstracts, editorials/letters, non-English language articles. (2) Full-text resources or raw data not available after contacting the original author; (3) sample size <10 cases; (4) follow-up time <30 days; and (5) study parameters that did not include outcome indicators of the inclusion criteria.

### 2.3. Data extraction and quality assessment

Two investigators independently performed the data extraction process. To reach a consensus, a third reviewer was consulted regarding possible inconsistencies during research selection, including the name of the first author, year of publication, sample size, age, success of implantation rate, follow-up time, indications for implantation, and extraction of the efficacy and safety indicators of HPCSP, such as NYHA cardiac function class, LVEF, LVEDd, QRS duration, pacing threshold, impedance, and complications at baseline and follow-up. Two investigators used the Newcastle-Ottawa (NOS) scale to rate the quality of nonrandomized research, which included the selection of study populations, comparability, and outcomes, The NOS evaluates studies using a star system (0–9). A study involving NOS ≥7 was deemed to be of high quality.

### 2.4. Statistical analysis

Random-effects models were used to analyze the data using Review Manager version 5.3. The results of the included studies were tested for heterogeneity by using the Q test: The study-specific magnitudes of effect and the heterogeneity (*I*^2^) statistics were used to measure statistical heterogeneity among studies for each outcome. A cut-off value of 50% was set for defining heterogeneity. *I*^2 ^≤ 50% implied that study heterogeneity was minor, and a fixed effect model was commonly used to describe the results; *I*^2 ^> 50% indicated that study heterogeneity is significant, and a random effect model was typically used to describe the results. Sensitivity analysis (mainly the method of eliminating article by article) or subgroup analysis was used to analyze the reasons for the large heterogeneity; if the heterogeneity was obvious (*I*^2 ^> 75%) or when sources of heterogeneity could not be found, descriptive analysis was used instead. The results of the meta-analyses are presented as forest plots. When the number of included literature was ≥9, publication bias could be assessed, and funnel plot analysis was performed at the same time. The funnel plot was evaluated for publication bias using Begg's and Egger's tests. In all studies, statistical significance was set at *P* < 0.05.

## 3. Results

### 3.1. Study and data selection

**Figure 1:**
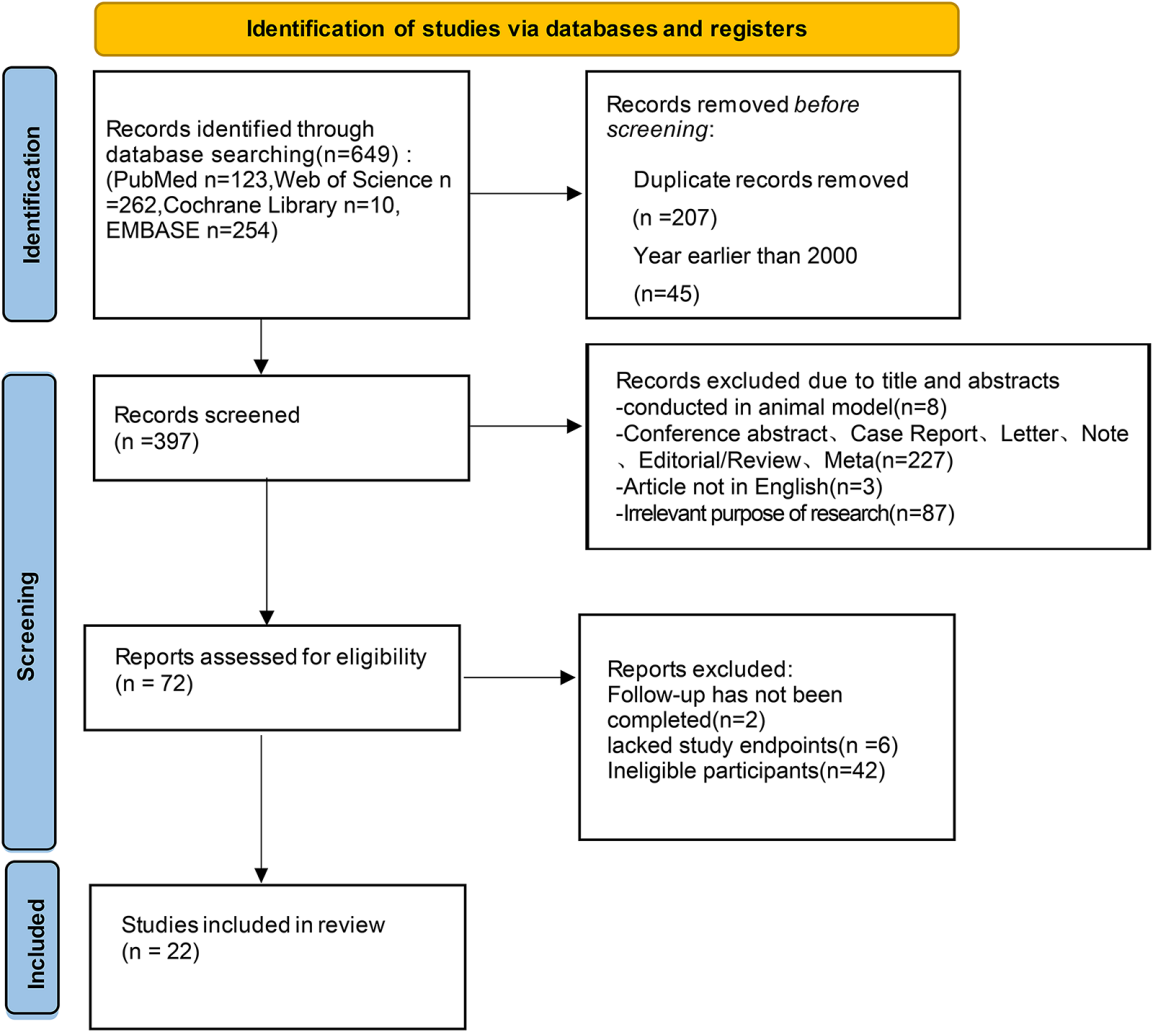
Flow diagram of study selection process.

A preliminary search of the database yielded 649 articles (123 articles from PubMed, 262 articles from Web of Science, 10 articles from the Cochrane Library, and 254 articles from Embase), and zero articles were retrieved manually. Based on the inclusion and exclusion criteria after screening, 22 studies on the treatment of patients with AF and HF using HPCSP were included. All patients met the indications for HPCSP. Figure 1 shows the literature-screening process and outcomes.

### 3.2. Quality assessment of included studies

The basic characteristics (name of first author, year of publication, study type, total number, age, implantation success rate, follow-up time, and pacing mode) of the 22 included studies were recorded. Table 1 presents the baseline and procedural features of the included studies. Two authors independently rated the RCTs and observational studies using the Newcastle-Ottawa (NOS) scale (Table 2), including high-quality literature. Subsequently, the full text of the articles was reviewed, and data were extracted. The extracted data included cardiac function class, LVEF, LVEDd, QRS duration, pacing threshold, and impedance.

**Table 1:** Procedural characteristics of included studies.

First author	Year	Study design	Sample size	Age (years)	Follow-up (months)	Pacing mode	Implant success (%)
Deshmukh et al. (16)	2000	Prospective study	18	69 ± 10	24	DHBP	66.70
Deshmukh and Romanyshyn (17)	2004	Prospective study	54	NR	42	DHBP	72.20
Occhetta et al. (18)	2006	Randomized crossover	18	71.4 ± 5.6	12	Para-Hisian pacing right apical pacing	88.90
Huang et al. (13)	2017	Prospective study	52	72.8 ± 8.3	20	HBP	80.80
Vijayaraman et al. (14)	2017	Retrospective study	42	74 ± 11	19 ± 14	HBP	95.00
Jastrzębski et al. (19)	2018	Retrospective study	125	73.0 ± 10.5	20.7 ± 15.0	HBP RVP	89.20
Wang et al. (20)	2019	Retrospective study	86	67.60 ± 10.85	37.1	HBP LBBP	94.50
Boczar et al. (21)	2019	Single-arm	14	67.35 ± 10	14.4	HBP	NR
Su et al. (22)	2020	Prospective study	94	70.1 ± 10.5	36	HBP	86.20
Žižek et al. (23)	2021	Retrospective study	24	68.8 ± 6.5	6	HBP BiV	100%
Moriña-Vázquez et al. (24)	2021	Prospective study	39	77 (70–81)	10.5 (3–12.5)	HBP	92.30
Li et al. (25)	2021	Retrospective study	72	59.1 ± 3.6	12	HBP RVP	NR
Ma et al. (26)	2021	Retrospective study	52	72.9 ± 10.9	17.06 ± 5.56	HBP BiV	88.10
Wu et al. (27)	2021	Prospective study	170	69.0 ± 10.1	12	HBP LBBP	95.50
Sheng et al. (28)	2021	Prospective study	26	72.9 ± 9.0	3	HBP LBBP	76.90
Ye et al. (29)	2021	Prospective study	16	NR	6	HBP LBBP	93.80
Yang et al. (30)	2021	Retrospective study	36	69.69 ± 13.75	11.52 ± 5.40	HBP LBBP	94.40
Pillai et al. (31)	2022	Retrospective study	98	75.8 ± 7.9 77 ± 6.7	36 ± 17 12 ± 8	HBP LBBP	94% vs 100%
Ivanovski et al. (32)	2022	Retrospective study	27HBP 10LBBP	71 (62–75) 69 (67–78)	6 2	HBP LBBP BiV	100
Vijayaraman et al. (33)	2022	Retrospective study	223	75 ± 10	27 ± 19	HBP LBBP BiV RVP	NR
Huang et al. (34)	2022	Randomized clinical trial	50	64.3 ± 10.3	18	HBP BiV	NR
Cai et al. (35)	2022	Prospective study	99	69.7 ± 9.7	12	HBP LBBP	100

**Table 2:** The Newcastle-Ottawa scale was used to assess the quality of the research included.

Study	Representativeness of the exposed cohort	Selection of the nonexposed cohort	Ascertain ment of exposure	Evidence indicating the desired outcome was absent at the beginning of the study	Comparability of cohorts on the basis of the analysis	Assessment of outcome	Was follow-up long enough for outcomes to occur	Was follow-up adequate In each group	Total scores
Deshmukh et al. (16)	*	*	*	*	*	*	*	*	8
Deshmukh and Romanyshyn (17)	*	*	*	*	*	*	*		7
Occhetta et al. (18)	*	*	*	*	*	*	*		7
Huang et al. (13)	*	*	*	*	*	*	*	*	8
Vijayaraman et al. (14)	*	*	*	*	*	*	*	*	8
Jastrzębski et al. (19)	*	*	*	*	*	*	*		7
Wang et al. (20)	*	*	*	*	*	*	*	*	8
Boczar et al. (21)	*	*	*	*	*	*	*	*	8
Su et al. (22)	*	*	*	*	*	*	*	*	8
Žižek et al. (23)	*	*	*	*	*	*			6
Moriña-Vázquez et al. (24)	*	*	*	*	*	*	*	*	8
Li et al. (25)	*	*	*	*	*	*	*		7
Ma et al. (26)	*	*	*	*	*	*	*	*	8
Wu et al. (27)	*	*	*	*	*	*	*	*	8
Sheng et al. (28)	*	*	*	*	*	*		*	7
Ye et al. (29)	*	*	*	*	*	*		*	7
Yang et al. (30)	*	*	*	*	*	*	*	*	8
Pillai et al. (31)	*	*	*	*	*	*	*	*	8
Ivanovski et al. (32)	*	*	*	*	*	*	*	*	8
Vijayaraman et al. (33)	*	*	*	*	*	*	*	*	8
Huang et al. (34)	*	*	*	*	*	*	*		7
Cai et al. (35)	*	*	*	*	*	*	*	*	8

### 3.3. Efficacy assessment

#### 3.3.1. Study characteristics

Of the 22 included studies, 10 were prospective, and 12 were retrospective cohorts. There of 1,445 patients were observed during follow-up, and the estimated implant success rate was 91.4%. The follow-up duration ranged from two to 53 months. The age of the study participants ranged from 55 to 85 years.

#### 3.3.2. Cardiac parameters

**Figure 2:**
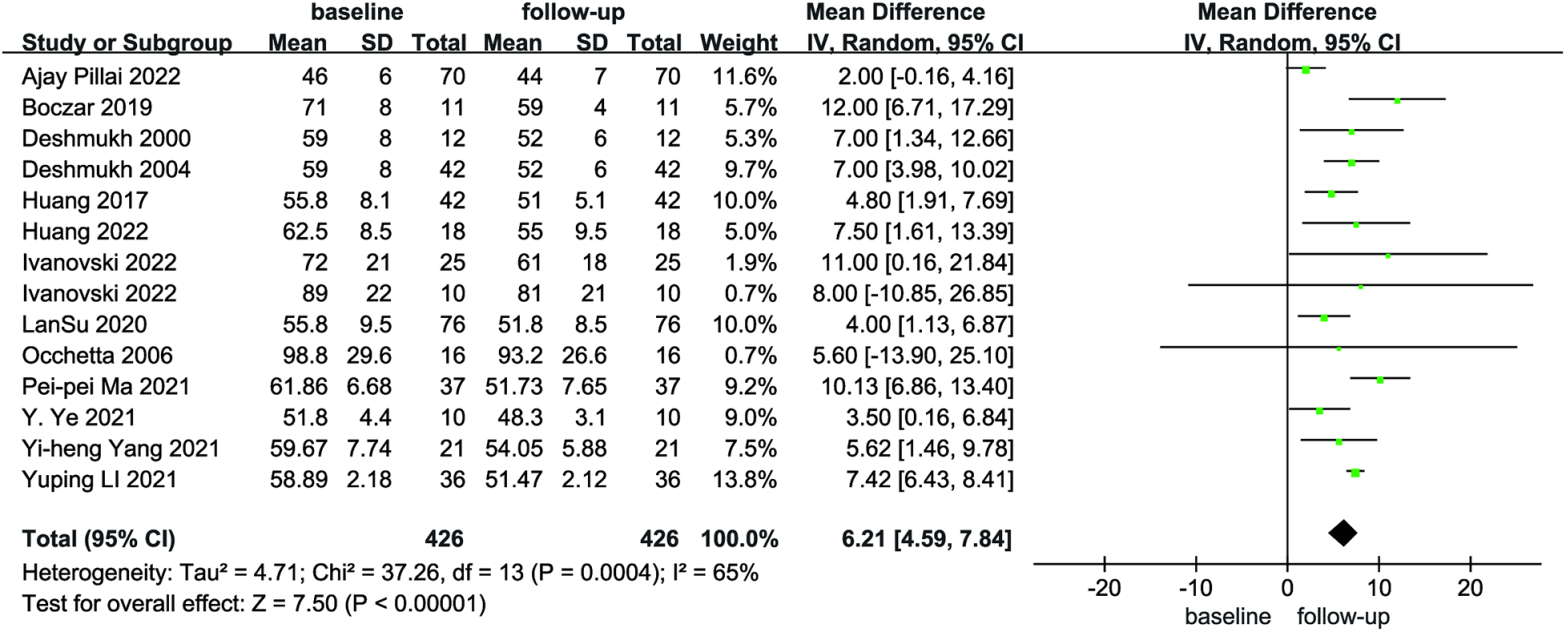
Forest plot of LVEDd for native vs. follow-up in the HPCSP group, LVEDd, left ventricular end-diastolic diameter; HPCSP, His-Purkinje system pacing.

**Figure 3:**
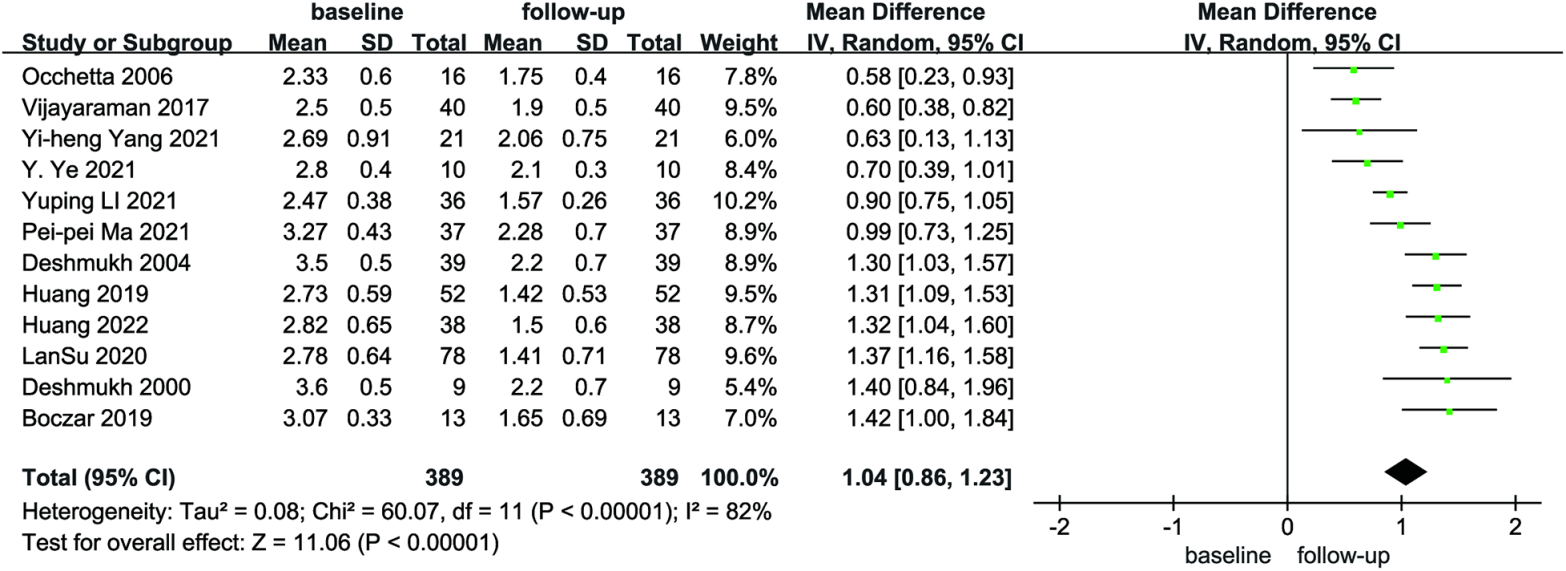
Forest plot of NYHA for native vs. follow-up in the HPCSP group, NYHA, New York heart association.

**Figure 4:**
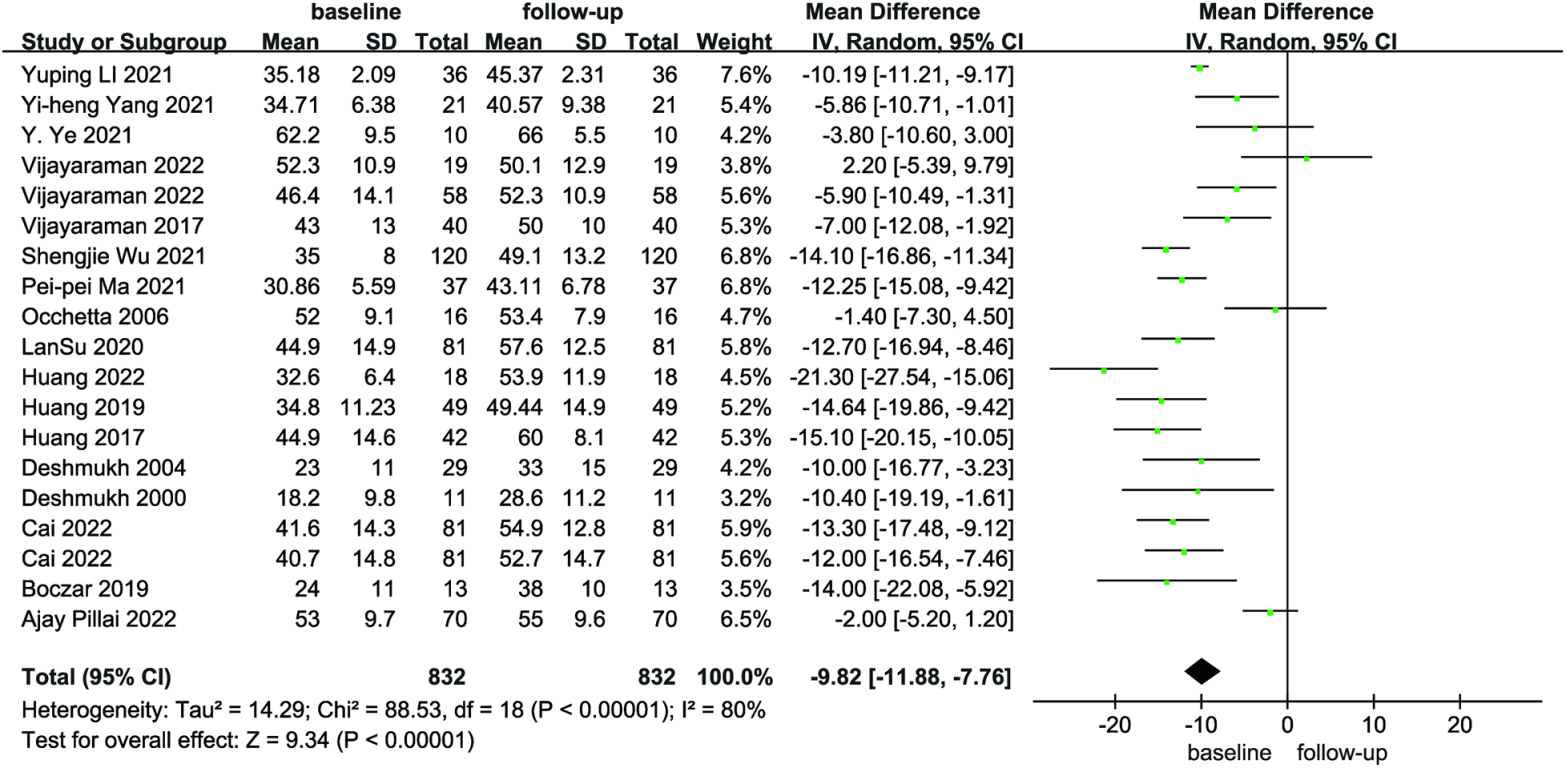
Forest plot of LVEF for native vs. follow-up in the HPCSP group, LVEF, left ventricular ejection fraction.

Compared with baseline, LVEDd (MD = 6.21, 95% CI: 4.59–7.84, *P* < 0.01) was significantly lower after follow-up (Figure 2). NYHA cardiac function class (MD = 1.04, 95% CI: 0.86–1.23, *P* < 0.01) was significantly decreased, as shown in Figure 3. We analyzed the LVEF of patients in 17 of these studies, which showed a considerable heterogeneity among them (*P* < 0.01, *I*² = 80%),the meta-analysis was conducted using a random effect model, and the results revealed that LVEF values increased by 9.82% (95% CI: −11.88–−7.76, *P* < 0.01 Figure 4).

**Figure 5:**
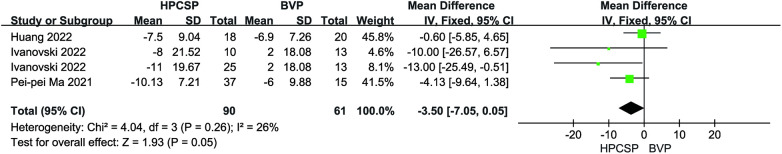
Forest plot of LVEDd for HPCSP vs. BVP, LVEDd, left ventricular end-diastolic diameter; HPCSP, His-Purkinje system pacing; BVP, biventricular pacing.

**Figure 6:**
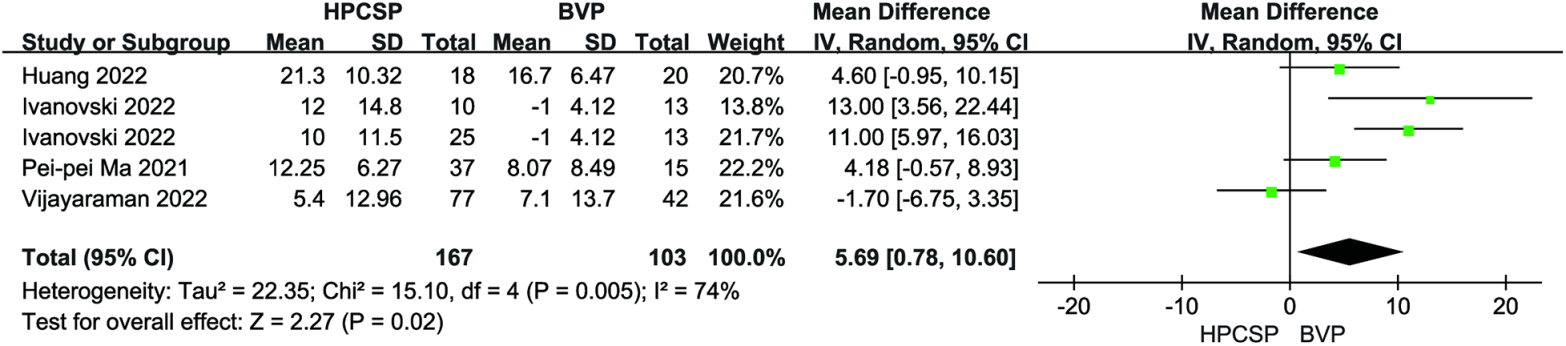
Forest plot of LVEF for HPCSP vs. BVP, LVEF, left ventricular ejection fraction; HPCSP, His-Purkinje system pacing; BVP, biventricular pacing.

Compared with the BiVP group, LVEDd decreased in the HPCSP group (MD = −3.50, 95% CI: −7.05–0.05, *P* = 0.05, Figure 5), the LVEF parameter in HPCSP group was superior to that of the BiVP group (MD = 5.69, 95% CI: 0.78–10.60, *P* = 0.02, Figure 6).

#### 3.3.3. QRS duration

**Figure 7:**
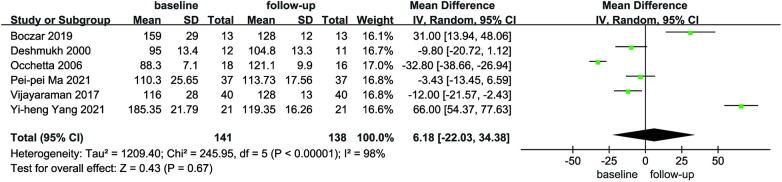
Forest plot of QRSd for native vs. follow-up in the HPCSP group, QRSd, QRS duration.

**Figure 8:**
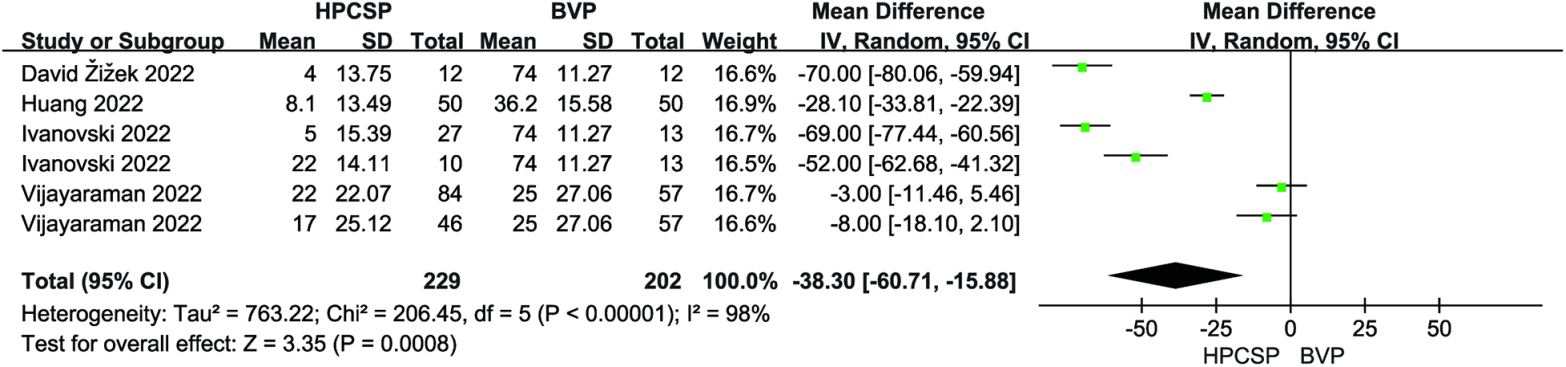
Forest plot of QRSd for HPCSP vs. BVP, QRSd, QRS duration.

Six articles reported the mean and standard deviation of QRS duration at baseline and follow-up. After combining and analyzing all data, there was no difference in QRS duration after follow-up compared with baseline in patients with AF combined with HF (MD = 6.18, 95% CI: −22.03–34.38, *P* = 0.67, Figure 7), the QRS duration was significantly shorter in HPCSP than those treated with BiVP (MD = −38.30, 95% CI: −60.71–−15.88, *P* < 0.01, Figure 8).

### 3.4. Safety assessment

#### 3.4.1. Pacing parameters

**Figure 9:**
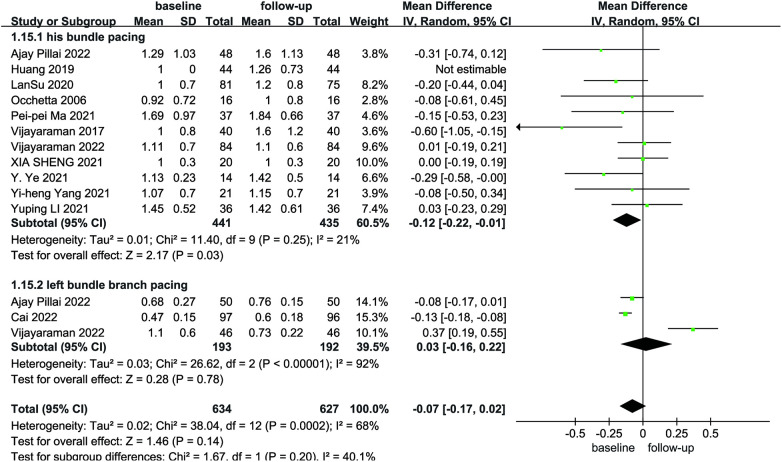
Forest plot of pacing threshold for baseline vs. follow-up in the HPCSP group.

**Figure 10:**
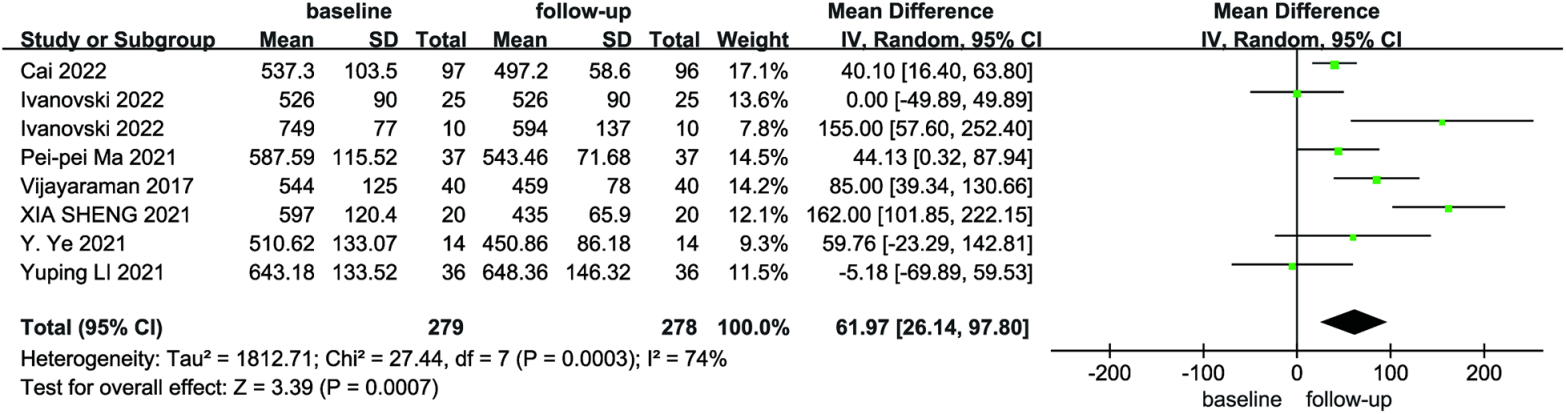
Forest plot of impedance for native vs. follow-up in the HPCSP group.

Due to the heterogeneity among the studies, a random-effects model was utilized for the pooled analysis of data on the initial and follow-up HPCSP thresholds (*P* < 0.01, *I*^2 ^= 68%). The findings showed no significant difference in pacing thresholds at follow-up compared with baseline (MD = −0.07; 95% CI: −0.17–−0.02; *P* = 0.14; Figure 9). The mean and standard deviation of impedance at baseline and after follow-up were reported in seven studies, which were analyzed using a random-effects model, considering the high heterogeneity (*I*² = 74%). Impedance decreases significantly after HPCSP compared with baseline (MD = 61.97; 95% CI: 26.14–97.80; *P* < 0.01; Figure 10).

#### 3.4.2. Endpoint events (all-cause death and rehospitalization)

**Figure 11:**
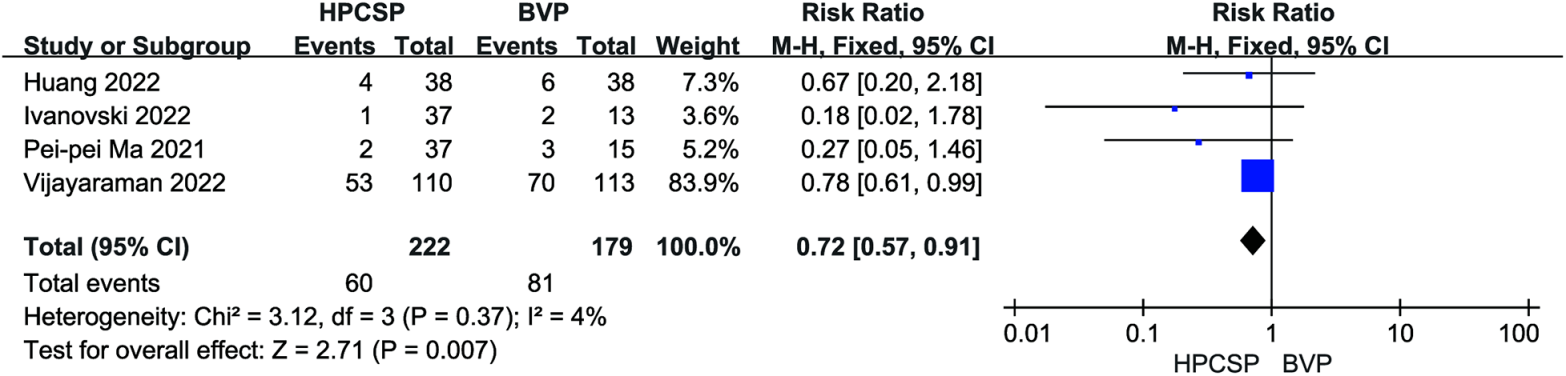
Forest plot of endpoint events for HPCSP vs. BVP.

A total of 8 studies documented endpoint events after the application of HPCSP. Application of BiVP increased the incidence of all-cause death and rehospitalization in patients compared to HPCSP (*I*^2 ^= 4%, *P* < 0.01, RR = 0.72, 95% CI: 0.57–0.91; Figure 11).

Among the 1,445 patients included in 22 studies, the overall success rate of pacing with the HPCSP was high, and at least one of the following 19 studies provided safety information. An increased pacing threshold (defined as a 1 V rise in capture threshold from the implant or a capture threshold > 5 V) was the most prevalent consequence, with 59 cases recorded. Lead repositioning was required in 6 patients, and lead dislocation or outlet obstruction was observed in 12 patients. Pouch infection occurred in 6 patients. There were two cases of ventricular septal perforation, three cases of left bundle branch loss and capture, and 20 cases of right bundle branch injury and complete atrioventricular block. During the follow-up period, 37 patients were re-hospitalized for HF. A total of 92 people died, 9 of whom died from CVD and the rest from non-cardiovascular causes.

### 3.5. Comparison between HBP and left bundle branch area pacing (LBBaP)

Seven studies in the included literature dealt with the comparison of LBBaP and HBP. The investigators compared the two pacing modalities in terms of the pacing threshold and endpoint events.

#### 3.5.1. Pacing threshold

**Figure 12:**
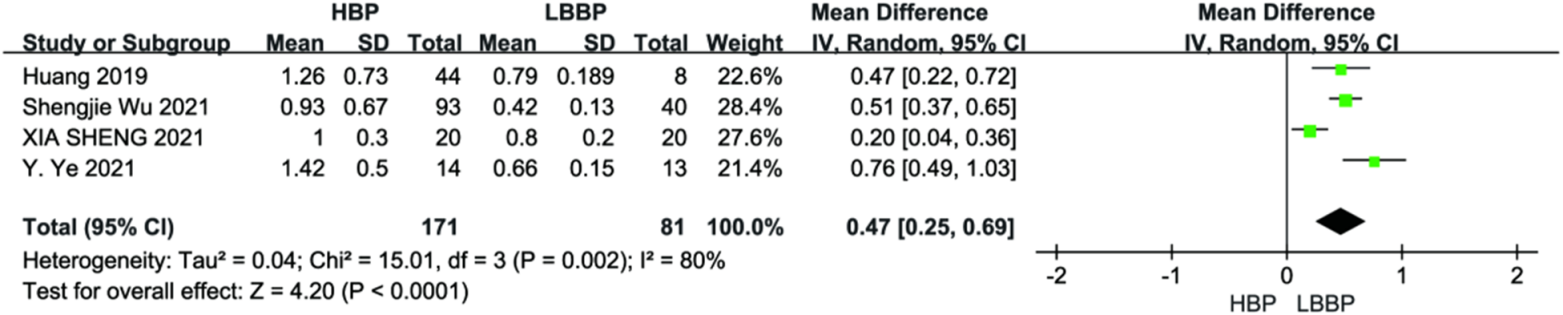
Forest plot of pacing threshold for HBP vs LBBP, HBP, his-bundle pacing; LBBP, left bundle branch pacing.

Four studies reported pacing thresholds during the procedure. A combined pooled analysis of the data showed that LBBP exhibited a lower pacing threshold than HBP during postoperative follow-up (MD = 0.47; 95% CI: 0.25–0.69; *P* < 0.01, Figure 12).

#### 3.5.2. Endpoint events

**Figure 13:**
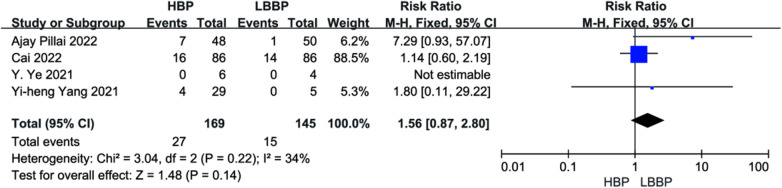
Forest plot of endpoint events for HBP vs. LBBP, HBP, his-bundle pacing; LBBP, left bundle branch pacing.

Four studies reported endpoint events. There was no effect on endpoint event rates compared with HBP and LBBP (*P* = 0.14, RR = 1.56, 95% CI: 0.87–2.80, Figure 13). The heterogeneity among these studies was low (*I*^2^ = 34.0%).

## 4. Discussion

The APAF-CRT study published at the 2021 ESC Annual Meeting provided a strong evidence-based medical basis that BiVP-CRT can maintain or improve cardiac function and significantly reduce the risk of death, and the results of this study promote the application of the Ablation + CRT strategy in patients with permanent AF and HF (36, 37). Although numerous observational studies suggest that BiVP maintains cardiac function better than RVP, as pacing strategies after AV node ablation have become more refined, experts have set the minimum threshold for the QRS interval at 130 ms. In actual clinical practice, 30% of patients do not respond. In addition, this pacing mode does not conform to the physiological pacing method because of drawbacks, such as increasing the QRS duration. Moreover, the use of coronary sinus electrodes in BiVP increases the complexity of placement, making electrode removal more difficult.

In contrast, HPCSP achieves physiological pacing by agitating the endogenous conduction system of the heart to avoid cardiac desynchronization and left ventricular dysfunction, thereby restoring the normal sequence of right and left ventricular excitation, and has received extensive attention from experts and scholars at home and abroad (38–40).

In this meta-analysis, 8 of these studies involved populations with slow heart rates that did not undergo AV node ablation, and the remaining 14 studies involved populations with fast AF heart rates that underwent AV node ablation combined with HPCSP. Relevant clinical studies were combined, and good cardiac echocardiographic indicators were observed, including the reduction of LVEDd, improvement of NYHA cardiac function class, and increase in LVEF value. The LVEF in the HPCSP group increased by an average of 9.82% from baseline to the last follow-up.

In 2000, Deshmukh et al. first reported the application of HBP in 18 patients with HF and AF undergoing AV node ablation, 12 of which were successful (16). Afterwards, many relevant clinical studies have been successfully conducted to confirm the feasibility and safety of the HPCSP in patients with AF and HF.

Ma et al. (26) first compared His bundle pacing with conventional biventricular pacing and found that 11 patients (29.73%) in the HBP group had a greater than 50% increase in left ventricular ejection fraction from baseline compared with only 1 patient (6.67%) in the biventricular pacing group, suggesting that His bundle pacing was more effective in improving cardiac function and delaying ventricular remodeling in these patients. The HIS-SYNC trial, a multicenter randomized study, found that HBP was superior to biventricular pacing for improving resynchronization in patients with HF. They predicted that HBP would be the preferred CRT strategy in future (41).

This meta-analysis included nine LBBP-related studies; we also performed a meta-analysis of LBBaP and HBP. After the follow-up period, the pacing threshold and impedance of the HBP group increased compared with baseline, while the LBBP group had an advantage over the HBP group in terms of impedance parameters. Huang et al. first reported the success of CRT in the left bundle branch region. After one year of follow-up, LVEF increased by 30% compared to baseline, LVEDd decreased by 34 mm, and the NYHA classification was upgraded from IV to I. These applications provide a clinical basis for the use of LBBP. The study found that LBBP was feasible in 97.8% of patients, maintained a low and stable pacing threshold, and had a low incidence of complications during follow-up (42). In 2021, Lan et al. evaluated the feasibility and safety of LBBP during long-term follow-up. This suggests that LBBP is expected to replace HBP and become a physiological pacing method with higher clinical feasibility (22). Due to the limited number of included populations, more large-scale multicenter randomized clinical trials are needed to explore the long-term outcomes of HBP and LBBP.

Synchronization of ventricular myocardial contractions and the order of conduction excitation are two important factors that affect cardiac function in patients after pacemaker implantation. The QRS wavewidth and morphology indirectly reflect the order of downstream excitation of the cardiac conduction bundle. Therefore, the QRS duration is an important indicator for judging the synchronicity of cardiac contraction (43), The abundant myocardial tissue around the leads ensures a low threshold for LBBP, which was also confirmed in our study. As illustrated by these stable pacing parameters, certain threshold or sensing problems common to BiVP or HBP can be avoided in LBBP (44). However, this meta-analysis has the following shortcomings. First, the number of patients collected in this systematic review was limited. Second, most of the included studies were observational studies, which have inherent limitations compared to randomized controlled studies. Third, Given the difficulties in properly performing conduction system pacing, the results could be influenced by the different approaches. Fourth, the study lacked verification of the 12-lead ECG after pacemaker implantation. Fifth, the data reported were from several different centers, and the criteria for echocardiographic metrics varied slightly from study to study, which may have an impact on the research results. In the actual application of HBP, attention should be paid to the pacing threshold and long-term lead performance. Compared with HBP, LBBP has better operability and safety; however, risks such as hematoma and perforation should also be considered when the lead is implanted.

In summary, as a more physiological pacing scheme, the HPCSP has gradually shown advantages in patients with HF and AF. Compared with BiVP, HPCSP significantly improved NYHA, increased LVEF, and lowered pacing thresholds. From a safety perspective, the HPCSP significantly reduces the occurrence of endpoint events. In addition, the cost of the HPCSP procedure is lower than traditional BiVP, making it more acceptable to patients and greatly improving patient compliance. Therefore, HPCSP is a better treatment option for patients with AF with HF who have failed repeated ablations and are not expected to have a high success rate of reablation. In this study, we found that LBBP can increase the QRS duration and carries the risk of right bundle branch injury and septal perforation, but the pacing parameters are better than those of HBP. Therefore, large prospective randomized controlled trials are needed to compare the long-term safety and clinical benefits of HBP and LBBP pacing modes.

## Data Availability

The original contributions presented in the study are included in the article/Supplementary Material, further inquiries can be directed to the corresponding author.
